# Effects of early-life cecal microbiota transplantation from divergently selected inbred chicken lines on growth, gut serotonin, and immune parameters in recipient chickens

**DOI:** 10.1016/j.psj.2022.101925

**Published:** 2022-04-22

**Authors:** Yuechi Fu, Jiaying Hu, Marisa A. Erasmus, Timothy A. Johnson, Heng-wei Cheng

**Affiliations:** ⁎ Department of Animal Sciences, Purdue University, West Lafayette, IN 47907, USA; † Livestock Behavior Research Unit, USDA-Agricultural Research Service, West Lafayette, IN 47907, USA

**Keywords:** cecal microbiota transplantation, chicken, intestinal architecture, immune parameter, serotonergic activity

## Abstract

Recent studies have revealed that fecal microbiota transplantation exerts beneficial effects on modulating stress-related inflammation and gastrointestinal health of the host. The aim of this study was to examine if cecal microbiota transplantation (**CMT**) presents similar efficiency in improving the health status of egg-laying strain chickens. Chicken lines 6_3_ and 7_2_ divergently selected for resistance or susceptibility to Marek's disease were used as CMT donors. Eighty-four d-old male recipient chicks (a commercial DeKalb XL layer strain) were randomly assigned into 3 treatments with 7 replicates per treatment and 4 birds per replicate (n = 7): saline (control, **CTRL**), cecal solution of line 6_3_ (**6_3_-CMT**), and cecal solution of line 7_2_ (**7_2_-CMT**) for a 16-wk trial. Cecal transplant gavage was conducted once daily from d 1 to d 10, then boosted once weekly from wk 3 to wk 5. The results indicated that 7_2_-CMT birds had the highest body weight and ileal villus/crypt ratio among the treatments at wk 5 (*P* ≤ 0.05); and higher heterophil/lymphocyte ratios than that of 6_3_-CMT birds at wk 16 (*P* < 0.05). 7_2_-CMT birds also had higher levels of plasma natural IgG and Interleukin (**IL**)-6 at wk 16, while 6_3_-CMT birds had higher concentrations of ileal mucosal secretory IgA at wk 5 and plasma IL-10 at wk 16 (*P* < 0.05), with a tendency for lower mRNA abundance of splenic IL-6 and tumor necrosis factor (**TNF)**-α at wk 16 (*P* = 0.08 and 0.07, respectively). In addition, 7_2_-CMT birds tended to have the lowest serotonin concentrations (*P* = 0.07) with the highest serotonin turnover in the ileum at wk 5 (*P* < 0.05). There were no treatment effects on the levels of plasma corticosterone and testosterone at wk 16 (*P* > 0.05). In conclusion, early postnatal CMT from different donors led to different patterns of growth and health status through the regulation of ileal morphological structures, gut-derived serotonergic activities, peripheral cytokines, and antibody production in recipient chickens.

## INTRODUCTION

Chickens reared in the large-scale commercial poultry production systems may experience various stressors, such as overcrowding, unstable social structure, transportation, and nutrient deprivation (Cheng et al., 2004; Matur et al., 2015). These risk factors drive pathophysiological changes in the gastrointestinal tract (**GIT**) (Konturek et al., 2011) and disrupt neuroendocrine and immune functions (Gensollen et al., 2016), resulting in decreased feed efficiency, poor health status, and economic losses in poultry (Li et al., 2017). To better fulfill the nutritional and health needs, the gut microbiota has emerged as a common intervention target for improving the production and welfare of farm animals (O'Callaghan et al., 2016). In humans, fecal microbiota transplantation (**FMT**) is an effective bacteriotherapy for treating recurrent *Clostridium difficile* infections and other gastrointestinal infectious diseases (Ianiro et al., 2020), with a potential for treating neuropsychiatric disorders (Cooke et al., 2021; Settanni et al., 2021). Similarly, FMT has been gradually applied to treat farm animals with various health issues, such as digestive disorders (inappetence and hypomotility) in ruminants (Mandal et al., 2017), resistance to African swine fever virus in pigs (Zhang et al., 2020), and post-weaning diarrhea in piglets (Ma et al., 2021). Hence, microbiota transplantation may have similar beneficial effects on the health and welfare of chickens.

In recent years, research on gut microbiota has gained great attention due to the essential contributions of microorganisms to host health across the host's lifespan (Rooks and Garrett, 2016). Emerging data suggest that intestinal microbiota can influence the functions of a variety of biological processes including the immune and neuroendocrine systems through the gut-brain and gut-immune axes, by which it impact host physiological and behavioral homeostasis (Marchesi et al., 2016; Verduci et al., 2020). Under normal circumstances, tight junction complexes connect the intestinal epithelial cells, forming a physical barrier to actively defend against invasions of pathogenic bacteria (Zhang et al., 2015). However, various sources of stress from the current intensive livestock production systems may damage the mucosa epithelial microstructures and increase gut permeability to toxins and pathogens, resulting in a pathophysiological syndrome, “leaky gut” in farm animals including chickens (Buffie and Pamer, 2013). Consequently, the damaged intestinal barrier increases the release of various proinflammatory cytokines into the blood circulation, causing systemic inflammation with activation of the hypothalamus-pituitary-adrenal (**HPA)** axis (Dinan and Cryan, 2012; Polansky et al., 2016) and increasing susceptibility to infectious diseases (Rychlik, 2020). An early study has suggested that serotonin (5-hydroxytryptamine, **5-HT**) interacts with the HPA axis functionally regulate pathophysiological homeostasis in humans and other animals (López et al., 1998). Serotonin (**5-HT**), as a neurotransmitter, is involved in mediating nutrient absorption, mental health, stress and immune responses (Ahern, 2011; Hestermann et al., 2014; Herr et al., 2017). However, the specific relationship between gut-derived 5-HT and stress-induced intestinal dysfunction is still under ongoing debate (Dong et al., 2017).

Early life has been increasingly recognized as a critical “window of opportunity” to modulate the gut microbiota due to its long-lasting effects on the host's biological homeostasis (Torow and Hornef, 2017; Sprockett et al., 2018). There are fluctuating changes in the gut microbial composition and diversity at an early age since gut colonization begins immediately after birth (Rodríguez et al., 2015). In newborn mammals, the first microbial encounter with maternal bacteria happens during passing through the birth canal, together with the bacteria within the local environment, contributing to the development of the baby's gut microbiota composition (Khoruts, 2016). Alteration in neonatal gut microbiota, such as early exposure to antibiotics before 6 months of age, contributes to an increased incidence of obesity in infancy and childhood (Trasande et al., 2013). Similarly, administration of probiotics 2 h after initiated incubation introduces beneficial effects to the embryonic development of broiler chickens (Baldwin et al., 2018). Therefore, ecological priority effects (early arrival of microbiota) play an important role in gut microbial development. On commercial poultry farms, chicks are from the fertilized eggs hatched in controlled environments without contact with adult hens. Therefore, it may provide an opportunity to transfer gut microbiota from adult birds to modify the biological characteristics of recipient chicks to improve their health and production performance. We hypothesized that similar to FMT in humans, early-life cecal microbiota transplantation may potentially improve immune and stress responses in chickens. Cecal contents were collected from two chicken lines, 6_3_ and 7_2_ which were divergently selected for resistance or susceptibility to Marek's disease, resulting in the line's unique physiological and behavioral characteristics. The birds of line 6_3_ are much gentler with higher egg production and lower social stress response than those of line 7_2_ (Bacon and Palmquist, 2002; Dennis and Cheng, 2014). The aim of this study was to investigate the effects of early-life cecal microbiota transplantation (**CMT**) from the divergently selected donors on performance traits, stress status, and immune characteristics in recipient chickens.

## MATERIALS AND METHODS

All procedures were approved by the Purdue University Animal Care and Use Committee (PACUC#: 1712001657) and the study was conducted in accordance with the guidelines set by the Federation of Animal Science Societies (2010).

### Birds and Experimental Design

Inbred chickens of the 6_3_ and 7_2_ lines developed at the Avian Disease and Oncology Laboratory (East Lansing, MI) were used as donors (Bacon et al., 2001). At 60 wk of age, the cecal content was randomly collected from 10 hens per line, then evenly pooled within each line. Five grams of pooled cecal contents were diluted 1:10 with gut microbiome media (adopted from Goodman et al., 2011), then kept at −20°C freezer until oral gavage.

A total of eighty-four 1-day-old male chicks (Dekalb XL, a commercial strain) were used as recipients and randomly allocated to 1 of 3 treatments with 7 cages per treatment and 4 birds per cage (n = 7): **CTRL** (0.1 mL saline, control), **6_3_-CMT** (0.1 mL cecal solution of line 6_3_), and **7_2_-CMT** (0.1 ml cecal solution of line 7_2_) for a 16-wk trial. Cecal microbiota transplant gavage was conducted once daily from d 1 to d 10, then boosted once weekly from wk 3 to wk 5. Water and feed were provided ad libitum. The general management, including vaccination, dietary formulation and nutrient contents, ambient temperature, and lighting program, was followed the Hy-line guidelines (2019).

### Sample Collection

At wk 5, 11, and 16, respectively, one bird per replicate was randomly selected for measuring body weight and blood sampling (n = 7). A 5 mL blood sample was collected from the brachial vein of each sampled bird using an **EDTA-coated** tube. After collection, the samples were centrifuged at 700 × *g* for 15 min at 4°C. Plasma was separated and stored at −80°C until further analysis.

The sampled birds were euthanized through cervical dislocation after blood sampling (n = 7). The liver, spleen, left adrenal gland, and heart weights were collected, then the spleen tissue samples from the same location of each bird were collected and frozen at −80°C for further analysis. In addition, approximately 7 cm of the ileum (near the diverticulum) per sampled bird was collected and flushed with sterile PBS to remove the contents, then separated into 2 parts: One part was immediately fixed with 10% buffered formalin and the other part was used for mucosal samples collection. The mucosal samples were scraped and collected, then frozen with liquid nitrogen and stored at −80°C.

### Blood Smear Analysis

At wk 16, the ratio of heterophils to lymphocytes (**H/L**) was measured from blood smears following a previously published protocol (Cheng et al., 2001b). One hundred heterophils and lymphocytes were counted from each slide (total 200 cells from 2 slides per bird) under a light microscope to determine the H/L ratio.

### Ileal Histomorphology

A 1-cm ileal specimen per bird was prepared as the procedure described by Jiang et al. (2020). Briefly, the formalin-fixed samples were dehydrated in graded ethanol solutions from 70 to 100%, cleared with xylene, then embedded in paraffin. Thereafter, 5.0-µm thick sections were sliced using a Leica RM 2145 microtome (Leica, Nussloch, Germany). The sections were stained with hematoxylin and eosin (Thermo, Waltham, MA), then examined using an Olympus BX40F-3 microscope (Olympus Cooperation, Tokyo, Japan). Three tissue sections containing intact lamina propria were selected from each bird, and an average of two readings (villus height, **VH** and crypt depth, **CD**, both measured in μm) were made from each section (total 6 counts per bird, 42 counts per group per time point). Image J software (NIH, Bethesda, MD) was used to measure VH and CD. The VH and CD per tissue sample were averaged, and the VH/CD ratio was calculated.

### HPLC

To determine the gut serotonergic activity, the ileal samples were analyzed in triplicate using HPLC (UltiMate 3000 RSLCnano System, Thermo Fisher Scientific Inc., Waltham, MA) as the procedure described by Yan et al. (2020). Briefly, the ileal samples were weighed and homogenized in 4 *M* perchloric acid at 1:5, then vortexed for 1 min. Afterward, the mixtures were centrifuged at 15,000 × *g* for 10 min at 4°C. The supernatants were drawn into a microcentrifuge tube and diluted with MD-TM mobile phase (Thermo Fisher Scientific Inc.) at 1:1. The mobile phase flow rate was 0.8 m/min. The ileal concentrations of 5-hydroxuindoleacetic acid (**5-HIAA**), 5-HT, and tryptophan were calculated as nanograms per gram of wet tissue (ng/g) using the relative reference curves generated from the corresponding calibrators.

### ELISA

Cecal microbiota transplantation-induced changes of plasma concentrations of Interleukin (**IL**)-6 (MBS037319, My BioSource, San Diego, CA), IL-10 (Catalog #: MBS007312, My BioSource, San Diego, CA), Tumor necrosis factor (**TNF**)-α (Catalog #: MBS260419, My BioSource, San Diego, CA), and IgG (Catalog #: E33-104, Bethyl Laboratories, Inc., Montgomery, TX) were measured using the respective ELISA kits following the relative company's instructions. Duplicate samples were taken with CV ≤15%.

Total protein levels in the ileal mucosal homogenates were measured by a Sigma Protein Assay kit (Sigma Chemical Co., St. Louis, MO) using bovine serum albumin as a standard (Dahlqvist, 1964). Mucosal secretory IgA concentrations were determined using a commercial ELISA kit (Catalog #: E33-103, Bethyl Laboratories, Inc.) following the manufacturer's guidelines. Concentrations of sIgA were expressed as micrograms of sIgA per gram of protein (mg/g).

### RIA

Total plasma concentrations of corticosterone and testosterone were determined in duplicate using commercially available I^125^ RIA kits (Catalog #: 07120103 and Catalog #: 07189102, MP Biomedicals, Solon, OH) as previously described (Cheng et al., 2001a). Briefly, 20 μL plasma was added to 80 μL diluents and then incubated at room temperature for 120 min. After the incubation, the tubes were vacated and the radioactivity was counted with a gamma counter (1470 Wizard Gamma Counter, PerkinElmer, Waltham, MA). The sensitivity of the assay was 0.02 ng/mL. All samples were assayed at the same time and duplicate samples were taken with CV ≤15%.

### RT-qPCR

Total RNA was extracted from the frozen spleen samples using RNeasy Mini Kit (Catalog #: 74804, Qiagen, Valencia, CA) following the instructions provided by the company. The purity and concentration of total RNA were checked using a NanoDrop 2000 (Thermo Scientific, Wilmington, DE). Reverse transcription was conducted using the Reverse Transcription Reagent Pack (Catalog #: N8080234, Applied Biosystems, Foster City, CA). A mixture of reverse transcription reagents consisted of 2 μL RNase inhibitor, 2.5 μL multi-scribe reverse transcriptase, 5 μL random hexamers, 10 μL of TaqMan reverse transcription buffer, 20 μL deoxynucleotides, and 22 μL of 25 mM magnesium chloride. A total mixture of each sample consisted of 61.5 μL with the adjusted volume of RNA sample and RNase-free water for a final 100 μL. The RNA samples were reverse transcribed to cDNA using a Techne TC-3000G PCR Thermal Cycler (Bibby Scientific Limited, Stone, UK). Splenic mRNA expressions of IL-6 (Assay ID #: Gg03337980_m1), TNF-α (Assay ID #: Gg03364359_m1), and IL-10 (Assay ID #: Gg03358689_m1) were detected by RT-qPCR using the primers and probes provided by its relative company. Glyceraldehyde 3-phosphate dehydrogenase (**GAPDH**) (Assay ID #: Gg03346982_m1, Applied Biosystems, Foster City, CA) was used as a reference gene. The PCR mixture contained 1.625 μL of TaqMan probe, 2.25 μL of gene-specific TaqMan forward and reverse primers each, 12.5 μL of PCR Master mix (Catalog #: 4304437, Applied Biosystems, Foster City, CA), 3.875 μL RNase-free water, and 2.5 μL of sample cDNA. The cycling conditions were 50°C for 2 min and 95°C for 10 min of the holding stage, followed by 40 cycles of 95°C for 15 s, then 60°C for 1 min. Results were quantitated by the standard curve method. Standards were measured in triplicates with a standard deviation of less than 2.0 and a coefficient of variation less than 2.0%.

### Statistical Analysis

Data were analyzed using R studio one-way ANOVA (version 3.6.2). The fixed effects were treatment and age. The Shapiro-Wilk test was used to analyze the normality of the data and non-normal data were logarithmically transformed. The Tukey-Kramer test was used to partition any significant differences among the least square means due to treatment main effects (Steel et al., 1997). Significance was set at *P* ≤ 0.05 and a trend difference was defined as 0.05 < *P* ≤ 0.10.

## RESULTS

### Performance Traits

**Figure 1:**
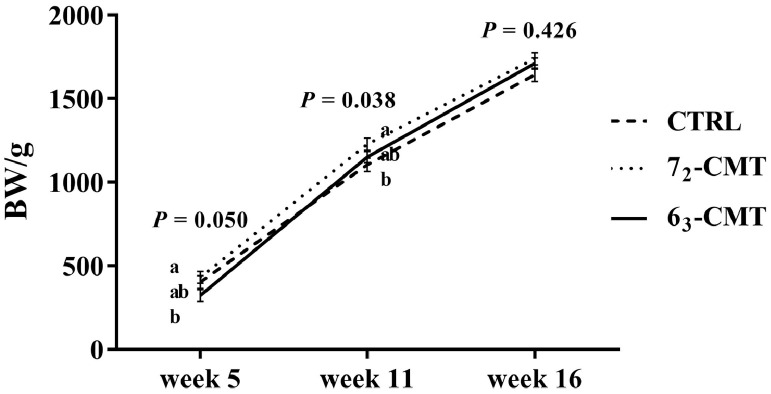
Effects of cecal microbiota transplantation on body weight of recipient roosters at 5, 11, and 16 wk of age. Values are least square means ± SEM, n = 7. ^a,b^ indicates significant differences (*P* ≤ 0.05). Abbreviations: 6_3_-CMT, birds with cecal bacterial solution of donor line 6_3_; 7_2_-CMT, birds with cecal bacterial solution of donor line 7_2_; CTRL, control.

**Figure 2:**
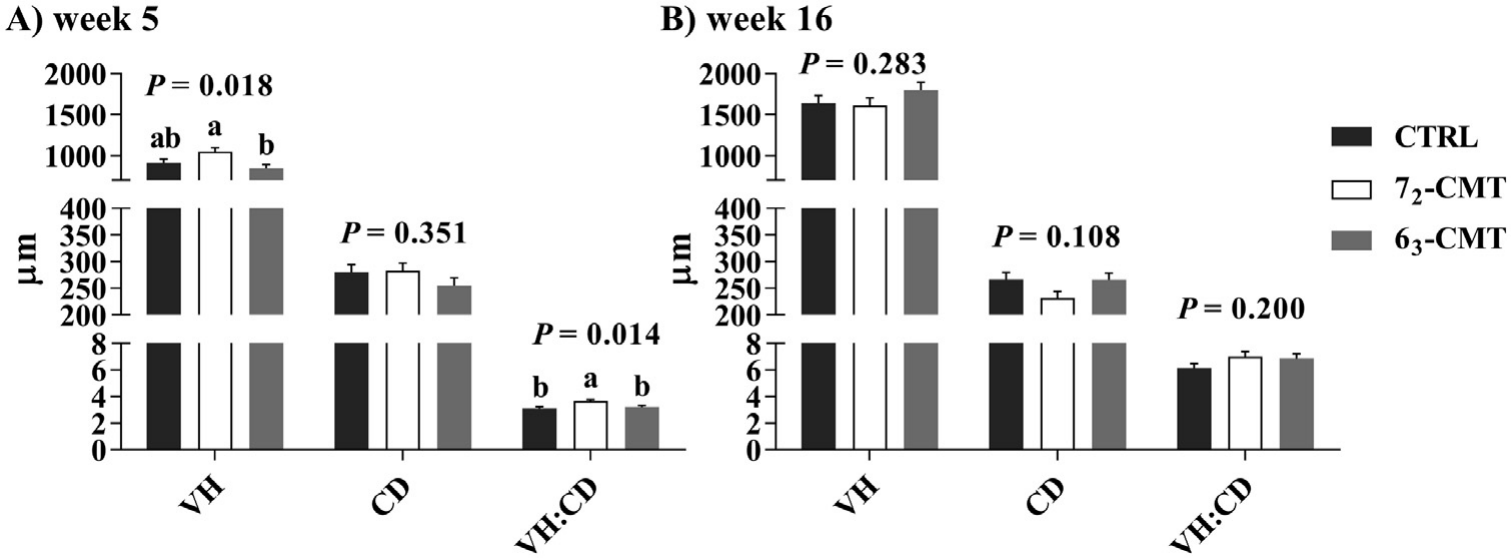
Effects of cecal microbiota transplantation on ileal morphology of recipient roosters at 5 and 16 wk of age. Ileal villus height (VH), crypt depth (CD), and VH/CD ratios at wk 5 (A) and at wk 16 (B). Values are least square means ± SEM, n = 7. ^a,b^ indicates significant differences (*P* ≤ 0.05). Abbreviations: 6_3_-CMT, birds with cecal bacterial solution of donor line 6_3_; 7_2_-CMT, birds with cecal bacterial solution of donor line 7_2_; CTRL, control.

Transplantation of cecal content from the divergently selected chicken donor lines differently affected physical and physiological characteristics of the recipient birds (Tables 1 and 2 and Figures 1 and 2). At wk 5, 7_2_-CMT birds had the highest BW (*P* = 0.050, Figure 1) among the recipient groups. The VH/CD ratio at wk 5 was also significantly higher in 7_2_-CMT birds than those of both 6_3_-CMT and CTRL birds (*P =* 0.014, Figure 2A). The differences were no longer present at wk 16 (*P* > 0.05, Figure 2B). In addition, 7_2_-CMT birds tended to have heavier relative adrenal glands than 6_3_-CMT birds (*P* = 0.090, Table 1) but not CTRL birds, while there were no treatment effects on the relative spleen, liver, and heart weights (*P* > 0.05). At wk 16, 7_2_-CMT birds had higher H/L ratios than 6_3_-CMT birds (*P =* 0.024, Table 2) but not CTRL birds, while no treatment effects were found on the concentrations of corticosterone and testosterone (*P* > 0.05).

**Table 1:** Effects of cecal microbiota transplantation on relative organ weights of recipient roosters at 16 wk of age.

Items	1Relative organ weight (%, g/kg)			
	Spleen	Liver	Adrenal gland	Heart
CTRL	184.938	1,799.055	4.181^AB^	637.256
7_2_-CMT	163.059	1,805.946	4.762^A^	666.078
6_3_-CMT	171.981	1,754.231	3.306^B^	711.921
SEM	13.421	94.099	0.420	66.227
*P*-value	0.524	0.923	0.090	0.748

Values are least square means ± SEM, n = 7.

Abbreviations: 6_3_-CMT, birds with cecal bacterial solution of donor line 6_3_; 7_2_-CMT, birds with cecal bacterial solution of donor line 7_2_; CTRL, control.

A,B Indicates trend differences (0.05 < *P* ≤ 0.10).

1 Relative organ weight = absolute organ weight (g)/ body weight (kg).

**Table 2:** Effects of cecal microbiota transplantation on stress parameters (H/L ratio, corticosterone) and sexual hormone (testosterone) of recipient roosters at 16 wk of age.

Measures	Treatment			SEM	*P*-value
	CTRL	7_2_-CMT	6_3_-CMT		
H/L ratio	0.327^ab^	0.367^a^	0.243^b^	0.029	0.024
Corticosterone (ng/mL)	4.235	4.678	3.697	0.900	0.789
Testosterone (ng/mL)	1.423	1.132	1.744	0.277	0.345

Values are least square means ± SEM, n = 7.

Abbreviations: 6_3_-CMT, birds with cecal bacterial solution of donor line 6_3_; 7_2_-CMT, birds with cecal bacterial solution of donor line 7_2_; CTRL, Control; H/L ratio, heterophil-to-lymphocyte ratio.

a,b Indicates significant differences (*P* ≤ 0.05).

### Immune Response

There were no treatment effects on the measured immune parameters among recipients at wk 5 (Table 3). At wk 11, 7_2_-CMT birds had higher levels of plasma IL-6 than CTRL birds (*P* = 0.002) and a tendency for higher plasma TNF-α than 6_3_-CMT birds (*P* = 0.091). These changes were continuously detectable at wk 16. Among the CMT recipient birds, 7_2_-CMT birds had greater concentrations of plasma natural IgG at wk 16 (*P* = 0.046). 7_2_-CMT birds also tended to have higher concentrations of plasma IL-6 (*P* = 0.070), while 6_3_-CMT birds had higher levels of plasma IL-10 (*P* = 0.045). In addition, 6_3_-CMT birds had higher concentrations of ileal mucosal sIgA at wk 5 (*P* = 0.045, Table 4). Consistent with these findings, 6_3_-CMT birds had a tendency of lower splenic IL-6 (*P* = 0.080) and TNF-α (*P* = 0.065) mRNA expressions than 7_2_-CMT birds at wk 16.

**Table 3:** Effects of cecal microbiota transplantation on levels of plasma natural IgG, pro- (IL-6 and TNF-α), and anti- inflammatory cytokines (IL-10) of recipient roosters at 5, 11, and 16 wk of age.

Treatment	IgG (mg/mL)	IL-6 (pg/mL)	TNF-α (pg/mL)	IL-10 (pg/mL)
5 wk of age				
CTRL	5.197	38.532	22.846	42.569
7_2_-CMT	5.412	37.109	26.495	33.259
6_3_-CMT	5.245	32.903	26.211	37.503
SEM	0.624	2.014	2.597	5.254
*P*-value	0.565	0.118	0.293	0.499
11 wk of age				
CTRL	8.486	32.562^b^	18.393^AB^	23.841
7_2_-CMT	10.830	41.713^a^	19.990^A^	23.724
6_3_-CMT	9.511	36.481^ab^	14.903^B^	20.855
SEM	1.335	1.465	1.571	2.853
*P*-value	0.363	0.002	0.091	0.709
16 wk of age				
CTRL	15.032^ab^	43.128^AB^	16.660	27.467^ab^
7_2_-CMT	17.993^a^	47.523^A^	21.706	26.928^b^
6_3_-CMT	13.716^b^	38.597^B^	16.161	33.835^a^
SEM	1.176	3.294	1.896	1.997
*P*-value	0.046	0.070	0.107	0.045

Values are least square means ± SEM, n = 7.

Abbreviations: 6_3_-CMT, birds with cecal bacterial solution of donor line 6_3_; 7_2_-CMT, birds with cecal bacterial solution of donor line 7_2_; CTRL, control; IL, interleukin; TNF-α, tumor necrosis factor alpha.

a,b Indicates significant differences (*P* ≤ 0.05).

A,B Shows trend differences (0.05 < *P* ≤ 0.10).

**Table 4:** Effects of cecal microbiota transplantation on mucosal sIgA concentrations and splenic relative mRNA abundance of pro- (IL-6 and TNF-α) and anti-inflammatory cytokines (IL-10) of recipient roosters at 5 and 16 wk of age.

Treatment	sIgA (mg/g)	Relative mRNA abundance		
		IL-6	TNF-α	IL-10
5 wk of age				
CTRL	2.167^ab^	0.806	0.905	0.396
7_2_-CMT	1.757^b^	0.763	1.378	0.461
6_3_-CMT	3.473^a^	0.673	1.280	0.258
SEM	0.440	0.141	0.175	0.153
*P*-value	0.045	0.796	0.296	0.456
16 wk of age				
CTRL	6.433	1.133^AB^	2.390^AB^	0.879
7_2_-CMT	7.989	1.694^A^	2.741^A^	0.739
6_3_-CMT	9.914	0.832^B^	2.217^B^	0.816
SEM	1.369	0.263	0.149	0.266
*P*-value	0.249	0.080	0.065	0.722

Values are least square means ± SEM, n = 7.

Abbreviations: 6_3_-CMT, birds with cecal bacterial solution of donor line 6_3_; 7_2_-CMT, birds with cecal bacterial solution of donor line 7_2_; CTRL, control; IL, interleukin; sIgA, secretory immunoglobulin A; TNF-α, tumor necrosis factor alpha.

a,b Indicates significant differences (*P* ≤ 0.05).

A,B Shows trend differences (0.05 < *P* ≤ 0.10).

### Ileal Serotonergic Activities

**Figure 3:**
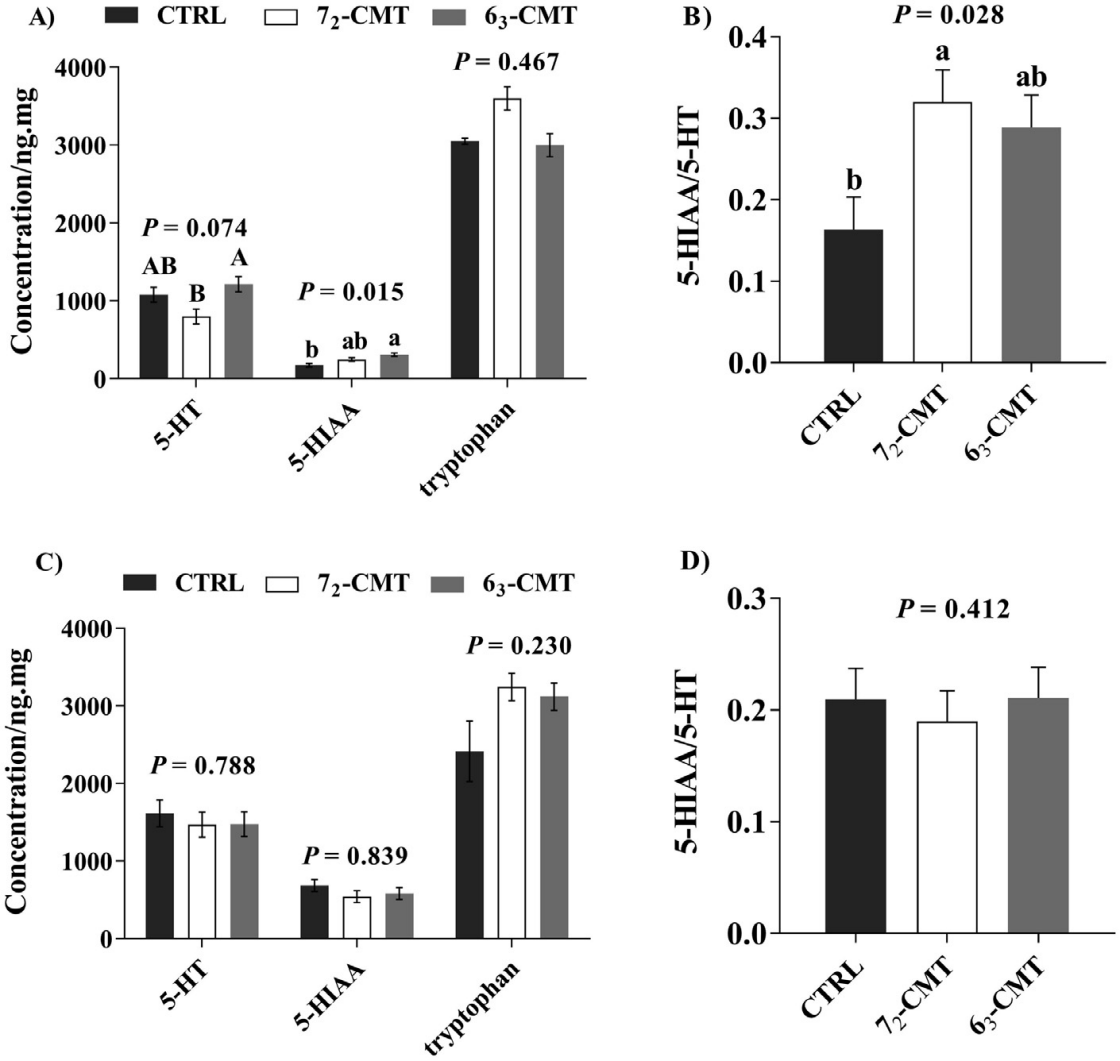
Effects of cecal microbiota transplantation on ileal serotonergic activities of recipient roosters at 5 and 16 wk of age. Serotonergic activity at wk 5 (A, B) and wk 16 (C, D). Values are least square means ± SEM, n = 7. ^a,b^ indicates significant differences (*P* ≤ 0.05), ^A,B^ shows trend differences (0.05 < *P* ≤ 0.10). Abbreviations: 5-HIAA, 5-hydroxuindoleacetic acid; 5-HT, serotonin; 6_3_-CMT, birds with cecal bacterial solution of donor line 6_3_; 7_2_-CMT, birds with cecal bacterial solution of donor line 7_2_; CTRL, control.

At wk 5, 6_3_-CMT birds had higher concentrations of 5-HIAA (*P* = 0.015) with a tendency of higher concentrations of 5-HT (*P* = 0.074, Figure 3A) in the ileum compared to both CTRL and 7_2_-CMT birds. There were no treatment effects on ileal tryptophan concentrations (*P* = 0.467). In addition, 5-HT turnover was higher in 7_2_-CMT birds as compared to CTRL birds at wk 5 (*P* = 0.028, Figure 3B). However, these treatment effects were undetectable at wk 16 (Figures 3C and 3D).

## DISCUSSION

### Cecal Microbiota Transplantation Alters Body Weight and Ileal Morphology in Recipient Chickens

One function of the gut microbiota is food digestion and nutrient absorption (Angelakis, 2017). In humans, patients with acute malnutrition can be treated with probiotic supplements to gain weight (Kerac et al., 2009). In our study, CMT led to BW changes in recipient birds, that is, 7_2_-CMT birds had the heaviest BW among the groups during the early growing phase (from day-old to 11 wk of age), the variations in weight gain among recipient birds may be associated with the changes in abundance of phyla *Firmicutes* in the gut. Recently, one of our studies revealed that 7_2_ donors have a higher abundance of *Firmicutes* (J. Hu, unpublished data) than 6_3_ donors, which is correlated with the heavier BW in 7_2_ birds (Dennis and Cheng, 2014). In supporting the hypothesis, previous studies reported that several members of the phyla *Firmicutes* function in energy resorption and production of short-chain fatty acids including butyrate (Ismail et al., 2011). Additionally, Million et al. (2013) found that germ-free (**GF**) mice receiving microbiota transplant from obese mice had a greater amount of fat content than those receiving microbiome from lean mice, which may be attributed to the enriched *Firmicutes*. Future studies are needed to examine how the transplantation-induced microbiome changes affect physical development in recipient birds.

In chickens, gut microbiota development is usually in a succession manner where the microbial community diversity is age-dependent (Rychlik, 2020). During a chick growth cycle, ongoing environmental exposures constantly change the gut microbial community and ultimately establish a relatively stabilized microbiota when reaching adulthood (Videvall et al., 2019). Together with the termination of boosting dosage at 5 wk of age, the similar BW among the recipient groups at wk 16 could be attributed to age- or treatment-related gut microbial stabilization. Additionally, Joat et al. (2021) reported that the gut microbiota composition in caged laying hens changed significantly from the rearing stage (pullets) to the production stage (layers) and the variations were mostly due to the differences in the management systems. In the current study, however, birds were maintained in the same growing facilities for the entire trial. As such, the unchanged BW in adult birds (roosters are sexually mature at approximately 16–20 wk of age) may be partially attributed to the evidence that functional core gut microbiota involved in the feed utilization has been stabilized at adulthood without continuous CMT boosting. In addition, weight gain requires sufficient nutrient absorption at an early age. Changes in the VH and CD have been commonly considered as key measurements for the assessment of gut maturation and nutritional effects. In line with the BW changes, we observed that 7_2_-CMT birds had the highest ileal VH and the greatest VH/CD ratios among the groups at wk 5. The ileum is the major absorption location for several nutrients such as vitamin B12 and fat in chickens (Mantle, 2020; Rupprecht and Bohórquez, 2021). Increased ileal VH may suggest that 7_2_-CMT birds have greater feeding digestion and nutrient absorption due to the enlarged epithelial surface areas at an early age (Caspary, 1992), while the treatment effects were reduced as the birds reach maturity. Collectively, early-life CMT affects growth performance through altering the BW and ileal microstructures in recipients, especially during early development.

### Cecal Microbiota Transplantation Influences Basic Stress Reactive Capability in Recipient Chickens

Hyperactivation of the HPA axis is commonly seen under multiple stress conditions, and corticosterone, as the final compound, is released from the adrenal glands within a short time following stimulation (Peirce and Alviña, 2019). However, the HPA axis is less developed in newly hatched chicks (Frankiensztajn et al., 2020). Generally, roosters become sexually mature at around 16 wk of age, which is a critical time point to assess reactively hormonal responses. Testosterone, as one of the sexual hormones in roosters, is synthesized by the testes under the regulation of both gonadotrophin and gonadotrophin-releasing hormones released from the pituitary and the hypothalamus, respectively (Ulloa-Aguirre and Timossi, 2000). The activation of the HPA axis often causes an inhibitory response of the hypothalamus-pituitary-gonad axis, resulting in a decreased level of testosterone (Tsutsui et al., 2012). In chickens, changes in the stress-related hormone, corticosterone, and stress indicators, such as H/L ratio has been considered as acute and chronic stress markers, respectively (Gross and Siegel, 1988; Cheng et al., 2001a; Kunz-Ebrecht et al., 2003). In the current study, CMT did not induce the differences in the basic levels of plasma corticosterone and testosterone among the treated birds at wk 16. These results agree with the previous studies that transplant of luminal contents from HF (high feather pecking) and LF (low feather pecking) selected chicken lines did not affect the levels of corticosterone in recipient birds (van der Eijk et al., 2020). Similar research conducted by Zhu et al. (2020) indicated that transplanting fecal content from either schizophrenic or healthy individuals did not alter the basic levels of corticosterone in recipient mice.

Recent advance in genetic technologies has unraveled the critical contributions of host genetics to the regulation of stress reactivity. For instance, the differences in stress adaptability are presented in the donor lines used in this study (Dennis et al., 2004). Line 7_2_ birds exhibit more aggressive behaviors than line 6_3_ birds in response to social stress, which may be associated with their variations in coping styles (reactive vs. proactive). Notably, we found that the basic stress response in recipient birds is correlated with those of the donors, reflected by a significantly lower H/L ratio together with a tendency of lighter adrenal gland weight in 6_3_-CMT birds as compared to 7_2_-CMT birds. In avian species, the change of adrenal gland weight has been considered as a chronic stress indicator (Harvey et al., 1984; Cheng et al., 2003). Generally, birds with heavier adrenal gland weight have greater adrenal activities in response to stress. Taken together, these results may suggest that cecal contents from the different donors differently influence stress responsible capability of recipient birds.

### Cecal Microbiota Transplantation Modulates Basal Immunity and Gut Health in Recipient Chickens

Extensive evidence has indicated that the crosstalk between the gut microbiota and immune system plays a vital role in maintaining the host's health status. Newly hatched birds, for example, are more susceptible to inflammation and infectious diseases due to a less developed gut microbial community as well as an immature immune system (Beal et al., 2005). In young birds, the innate immune system constitutes the first line of the defense system protecting against pathogenic infections or inflammation induced by environmental stressors (Bar-Shira and Friedman, 2006). In adulthood, the intestinal microbiota affects the recruitment of immune cells, activating both antibody-dominated and cellular immune responses (Dempsey et al., 2003; Broom and Kogut, 2018). To better understand how the CMT impacts the immune system, we examined the changes of plasma concentrations of circulating natural antibody (IgG), proinflammatory cytokines (IL-6 and TNF-α), and anti-inflammatory cytokine (IL-10) at 5, 11, and 16 wk of age. Interestingly, 7_2_-CMT birds had higher levels of plasma natural IgG compared with 6_3_-CMT birds during their sexual maturity at 16 wk of age. Natural IgG, as one of the most abundant antibodies, presents in the circulation after birth even in the absence of prior exposure to a defined antigen (Casali and Schettino, 1996). The increased levels of natural IgG in humans have been linked to the pathological processes in disease-induced tissue and cell damage or a breakdown in the host's self-tolerance (Nagele et al., 2013). In humans, organ-specific or systemic autoimmune diseases could be aggravated by the increased binding of self-reactive IgG with the targeted tissues, organs, or free molecules including phospholipids (Elkon and Casali, 2008; Nimmerjahn and Ravetch, 2021). In the current study, recipient birds were exposed to similar environmental conditions and under the same management practices, higher concentrations of natural IgG in 7_2_-CMT birds may be explained by exaggerated immunological responsiveness to social and environmental stressors resulting from group-housed in cages. This view is supported by the susceptibility of donor line 7_2_ to Marek's disease and exhibition of greater aggressiveness in response to social challenges (Dennis and Cheng, 2014). Together with the genetic-microbiota interaction, the transferred bacteria from the donor line 7_2_ may induce greater production of autoantibody IgG in 7_2_-CMT birds. Accumulating evidence has indicated that genetic variability of animals leads to different immune response (Cheng et al., 2001b; Parmentier et al., 2004; van der Eijk et al., 2019). For instance, in the donor lines, 7_2_ birds have higher concentrations of serum IgG as compared with line 6_3_ (Bacon and Palmquist, 2002). Although it is unclear how CMT (i.e., what are the transferred bacteria) affects IgG synthesis in recipient birds, previous studies have reported that the genus *Lactobacillus* is enriched in both the caeca and feces of chickens infected with Marek's disease virus (Perumbakkam et al., 2014). Due to the CMT, 7_2_-CMT birds may develop a similar pattern of gut microbial composition as those seen in 7_2_ donors, including the enriched genus *Lactobacillus*, by which it may induce great IgG production (Perumbakkam et al., 2016). These results reveal that donors’ genotype and gut microbiota may work together to influence the immunity of recipient birds.

Maintenance of intestinal homeostasis requires appropriate discrimination between beneficial and pathogenic bacteria (Yoo et al., 2020). Mucosal secretory (s)IgA, as one of the most abundant antibodies within the intestinal lumen, protects the gut epithelia from invading pathogenic bacteria and related tissue damage (Mantis et al., 2011). Mucosal sIgA has been used as a biomarker for evaluating intestinal homeostasis (de Santis et al., 2015). As expected, our results showed the ileal mucosal sIgA levels were significantly affected by CMT at wk 5. The higher levels of sIgA in 6_3_-CMT birds may help them maintain gut health as well as improve nutrient absorption. Considering the protective roles that mucosal sIgA plays in the intestinal barrier, sIgA may directly and indirectly mediate cytokine production. Brzozowski et al. (2016) reported that the breakdown of the intestinal epithelial layer is associated with the changes in mRNA abundance and synthesis of inflammatory cytokines. In our study, 7_2_-CMT birds had attenuated levels of an anti-inflammatory cytokine, IL-10, but greater concentrations of proinflammatory cytokines, IL-6 and TNF-α, among the groups. In line with this finding, CMT tended to decrease mRNA abundance of proinflammatory cytokines, such as IL-6 and TNF-α, in the spleen of 6_3_-CMT birds, which may suggest that transferred cecal microbiota from different donor birds differently affects the immunity of recipient birds. These results further reveal the critical role of the gut microbiota in regulating gut health and immune response in chickens.

### Cecal Microbiota Transplantation Affects Gut-Derived Serotonin in Recipient Chickens

The GIT is the major location of peripheral 5-HT, approximately 95% of a body's 5-HT is synthesized by the gut mucosal enterochromaffin cells (Racké et al., 1989; Banskota et al., 2019). Subsequently, investigations have uncovered a range of functions of gut-derived 5-HT, including regulation of gut motility, secretion of bioactive factors (Mawe and Hoffman, 2013), metabolic processes (Jones et al., 2020), and bone formation (Yadav et al., 2009; Sjögren et al., 2012). Further, gut-derived 5-HT acts on the activation of immune cells via signaling a variety of 5-HT receptors, which in turn regulates cytokine production (de Haas and van der Eijk, 2018; Liu et al., 2021). Given its multiple roles, the changes of gastrointestinal 5-HT may have implications for inflammatory signaling and stress response. Here, we found that 6_3_-CMT birds had higher concentrations of 5-HIAA, a metabolite of 5-HT, with a tendency for higher concentrations of 5-HT than 7_2_-CMT birds at wk 5. These results may suggest that 6_3_-CMT birds have a more activated serotonergic system than 7_2_-CMT birds. In addition, 5-HIAA has been used as a biological marker for predicting inflammatory conditions (Croonenberghs et al., 2000; Dwarkasing et al., 2016; Jayamohananan and Kumar, 2019). Imbalanced 5-HT synthesis promotes the pathological process of stress-induced diarrhea in mice (Dong et al., 2017). Margolis et al. (2014) also suggested that 5-HT can modulate gut physiology by facilitating gut inflammation. In our study, the high activated gut serotonergic systems in 6_3_-CMT birds were paralleled by higher concentrations of mucosal sIgA and plasma IL-10, which may indicate the transferred microbiota induces anti-inflammatory effects in recipients. Therefore, these changes may indicate that CMT could be a potential method to increase protective intestinal immunity in chickens.

Previous studies have reported that chronic stress results in a decrease of mRNA abundance of TPH1 in the intestines, an enzyme for the synthesis of peripheral 5-HT (Yue et al., 2017). In agreement with this finding, Coates et al. (2004) suggested that stress altered gut-derived 5-HT signaling, thereby downregulating 5-HT levels and TPH1 mRNA abundance in the colon, leading to various gut disorders including irritable bowel disease. This hypothesis is supported by the fact that oral tryptophan supplementation, the precursor of 5-HT, reduces experimental non-alcoholic fatty liver disease in mice by ameliorating the dysregulated intestinal serotonergic system and stabilizing the intestinal barrier (Ritze et al., 2014). Keszthelyi et al. (2014) also reported that administration of 5-hydroxytryptophan (5-HTP), the intermediate metabolite of tryptophan, promotes the production of tight junction proteins and reduces gastrointestinal mucosal permeability. Although the functions of gut serotonergic activity in stress response are not examined in the current study, high levels of gut serotonergic activity in 6_3_-CMT birds may imply that they have better stress adaptive capability. The results provide a sign for future studies to verify the functional role of gut-derived 5-HT in regulating stress reactions in chickens.

## CONCLUSIONS

This study demonstrates that early postnatal CMT influences growth, gut morphological development, immunity, and stress adaptive capability of recipient chickens via the microbiota-donor-host interactions. The results indicate that microbiota transplantation, especially at an early age, could be a novel strategy for ameliorating stress response and improving chicken health and welfare status. Future studies are needed to investigate the potential associations between specific beneficial bacterial taxa and physiological and behavioral characteristics in the donor-recipient relationship, which could provide a novel management strategy for poultry production.
